# Different maturation of gut microbiome in Korean children

**DOI:** 10.3389/fmicb.2022.1036533

**Published:** 2022-11-23

**Authors:** Jieun Kim, Erin Kim, Bongyoung Kim, Jinsup Kim, Hyun Ju Lee, Jun-Sun Park, Sehee Hwang, Mina Rho, Hyunjoo Pai

**Affiliations:** ^1^Department of Internal Medicine, College of Medicine, Hanyang University, Seoul, South Korea; ^2^Department of Computer Science and Engineering, Hanyang University, Seoul, South Korea; ^3^Department of Clinical Development, Novel Pharma Inc., Seoul, South Korea; ^4^Department of Pediatrics, College of Medicine, Hanyang University, Seoul, South Korea; ^5^Translational Research Center, Research Institute of Public Health, National Medical Center, Seoul, Republic of Korea; ^6^Cancer Information Center, Yonsei University Health System, Yonsei Cancer Center, Seoul, South Korea; ^7^Department of Biomedical Informatics, Hanyang University, Seoul, South Korea

**Keywords:** gut microbiome, bacterial composition, antibiotic resistance genes, children, adult

## Abstract

**Introduction:**

Gut microbiome plays a crucial role in maintaining human health and is influenced by food intake, age, and other factors.

**Methods:**

In this study based in Korea, we examined the bacterial taxonomic composition of the gut microbiota in infants (≤ 1 year), toddlers (1–<4 years), and school-aged children (4–13 years) and compared them with those of healthy adults to investigate the microbiota changes in early life and their association with the resistome. We used whole metagenome sequences obtained by Illumina HiSeq sequencing and clinical information of 53 healthy children, and sequence data of 61 adults from our previous study.

**Results:**

Our results indicate that the bacterial proportion of the gut in the population ranging from infants to adults forms three clusters: the *Ruminococcus-Eubacterium* (G1), *Bifidobacterium-Escherichia* (G2), and *Bacteroides-Faecalibacterium* (G3) groups. The gut microbiota of infants and toddlers (100% of infants and 85% of toddlers) constituted mostly of G2 and G3 groups, whereas 90% of adults showed G1-type gut microbiota. School-aged children showed a transitional gut microbiota composition of both infants and adults (31%, 38%, and 31% in G1, G2, and G3, respectively). Notably, the three clusters of microbiota showed significantly different patterns of bacterial diversity (*p* < 0.001): G2 showed the lowest Shannon index, followed by G3 and G1 (1.41, 2.08, and 2.48, respectively; median Shannon index). When combined with the adult group, alpha diversity showed a positive correlation with age (R^2^ = 0.3). Furthermore, clustering the composition of antibiotic resistance genes (ARG) identified two clusters (A1 and A2), and most of G1 (95%) and G3 (80%) belonged to A1. However, G2 showed the least diversity and the highest abundance of ARGs. Nine ARG families showed a significant difference among age groups; three tetracycline resistance genes, *tet32*, *tetO*, and *tetW*, showed a positive correlation, and six other genes, *ampC*, *TEM*, *ileS*, *bacA*, *pmr transferase*, and *cepA*, showed a negative correlation with age.

**Discussion:**

In conclusion, our results highlighted that a delayed persistence of the *Bifidobacterium*-dominant enterotype with a lower bacterial diversity was observed in Korean children up to 13 years of age, which suggests a different maturation process with a delayed maturation time.

## Introduction

The human gut microbiota plays a critical role in human health, including metabolism and immunity (Qin et al., 2012; Kurilshikov et al., 2017). Gut microbiota are established from birth and shaped during the first few years of life, and a deviation in this development may have consequences in later life (Derrien et al., 2019). The developing gut microbiota in infants undergoes distinct phases of progression: development, transitional, and stable phases (Stewart et al., 2018). In this transition of gut microbiota in infants, the diversity of bacterial proportion increases, the structure is stabilized, and the bacterial dominance changes from *Firmicutes* and *Bifidobacterium* to *Bacteroides* and *Prevotella* (Xiao et al., 2021). Multiple clinical factors are associated with the distribution of enterotypes during the development of gut microbiota in infants (Derrien et al., 2019; Xiao et al., 2021). The geographic location was one of them; enterotype distribution in infants differed according to the population of different countries (Xiao et al., 2021). For example, the *Bifidobacterium*-dominant enterotype persisted until late infancy in several Asian and South American countries, unlike in European countries, where the infant microbiota showed a clear transition to *Bacteroides* and *Prevotella* enterotypes (Xiao et al., 2021). In Korea, *Bifidobacterium* was abundant in the healthy adult population, as shown in our previous study (Cho et al., 2022). Therefore, it is necessary to study the region-wise development of gut microbiota in infants and children as they may exhibit a different gut bacterial proportion.

The microbiome taxonomic composition and postnatal age influence both the overall resistome and individual antibiotic resistance genes (ARGs) in the general infant population (Bäckhed et al., 2015; Gibson et al., 2016; Lebeaux et al., 2021). Previous studies have documented that the relative abundance of ARGs was greater in younger infants than in young children or adults, and the types of ARGs varied among age groups (Bäckhed et al., 2015; Gibson et al., 2016; Lebeaux et al., 2021). These trends occurred in parallel with the taxonomic composition development within the gut of infants (Lebeaux et al., 2021), and several individual early life factors, such as delivery mode, perinatal antibiotic use, and breast or formula feeding, shape the bacterial proportion and resistome in infants (Bäckhed et al., 2015; Gibson et al., 2016; Lebeaux et al., 2021; Li et al., 2021; Xiao et al., 2021; Lebeaux et al., 2022).

Therefore, we examined the bacterial taxonomic composition of the gut microbiota in infants (≤1 year), toddlers (1–<4 years), and school-aged children (4–13 years) in Korea and compared them with those of healthy adults to understand the gut microbiome dynamics of developing children in Korea. In addition, we compared the characteristics of the resistome in each age group and analyzed the association between resistome and enterotypes.

## Materials and methods

### Study design and sample collection

This study was conducted using healthy people who visited the Hanyang University Hospital in South Korea. The study protocol was approved by the Institutional Review Board (HYUH IRB 2019–09-030) and written informed consent was obtained from all the participants. Written informed consent to participate in this study was provided by the participants’ legal guardian/next of kin.

**Figure 1:**
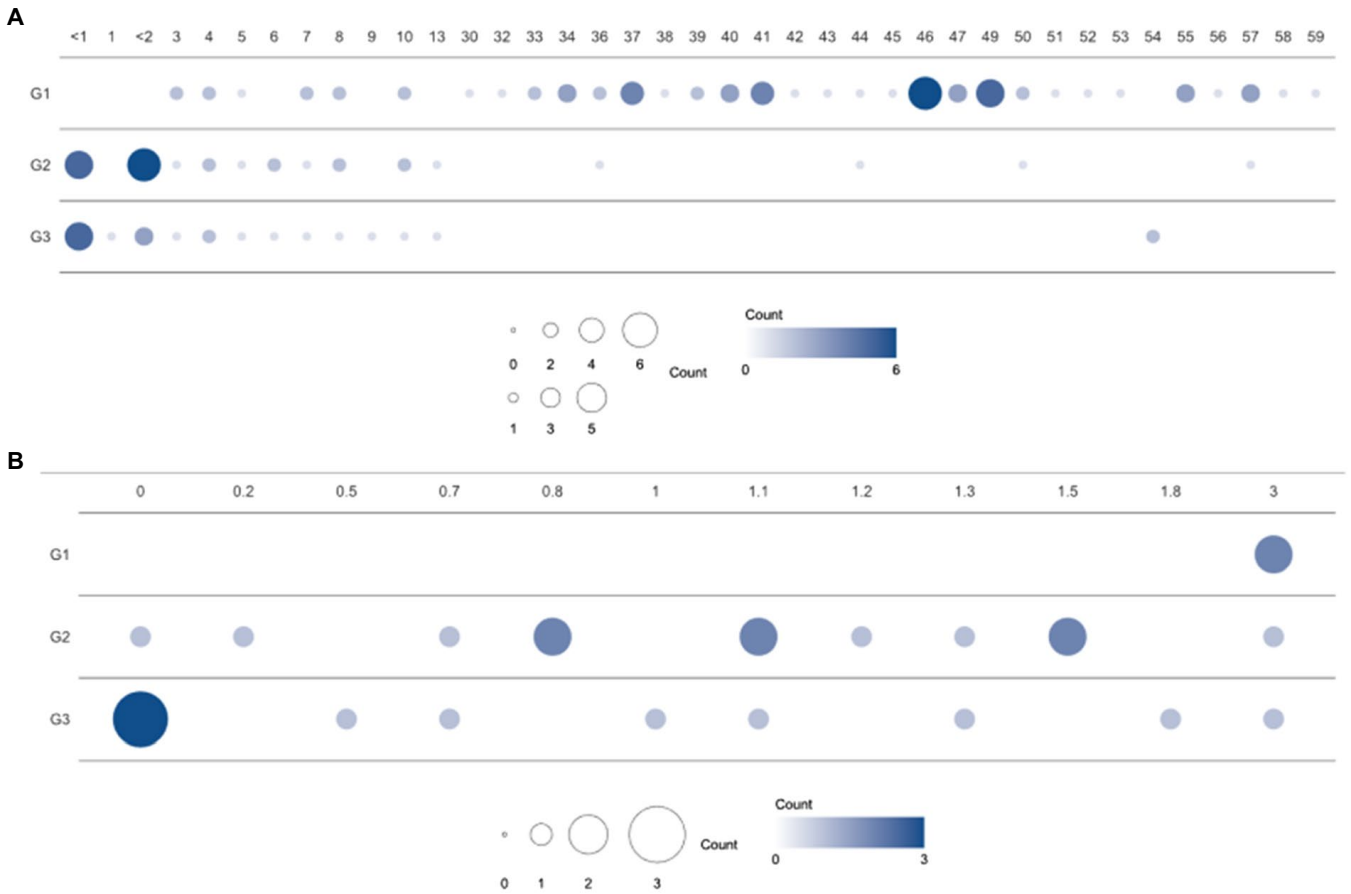
Population distribution over three enterotype clusters and ages. **(A)** Number of samples from individuals of each age in the clusters (G1, G2, and G3) based on bacterial proportion and **(B)** using child samples aged 3 years or under. G1, G2, and G3 were from Dirichlet multinomial mixture (DMM) clustering using bacterial proportion.

A total of 114 metagenome samples were used: 53 from healthy infants and children (1–13 years) and 61 from healthy adults (Figure 1). Sequencing data from healthy adults were obtained from our previous study (Kim et al., 2021). Healthy adults aged 30–59 years, with a Charlson comorbidity score of zero, who visited health screening services from June–October 2017 were sampled for this study (Kim et al., 2021). Faecal samples were collected from healthy infants and children aged <13 years enrolled in the national immunization program between October–December 2019. Healthy infants and children were defined as those without any medical conditions other than allergic disease. A questionnaire about personal information was used to collect the data for infants and children. Personal information included delivery mode, birth weight, gestational age, and breastfeeding duration. The questionnaire also contained the medical history of the year prior, such as hospitalization, frequency of hospital visits, and use of antibiotics. About 30–50 g of faecal sample was collected from each individual into a sterile container and stored frozen at −80°C prior to extraction of total DNA content.

### Faecal DNA preparation, sequencing, and sequence filtering

Total DNA was extracted using the Fast DNA SPIN Kit for Faeces (MP Biomedicals, #116570200, CA, USA), following the manufacturer’s instructions. The Illumina HiSeqX Platform (Illumina, San Diego, CA, USA) was used to sequence the DNA samples. For every sample, 151 bp paired-end sequences were generated from inserts of 350 bp. Low-quality reads were removed using Sickle (Joshi and Fass, 2011), and reads containing ‘N’ were also removed. Finally, host contamination was removed by discarding reads mapped to the human genome, provided by the National Center for Biotechnology Information (NCBI).

### Profiling of microbial composition and antibiotic resistance genes

The program MetaPhlAn (version 2.0; Truong et al., 2015) was used to classify microbial community composition using the default option. In the MetaPhlAn process, bowtie2 (Langmead and Salzberg, 2012) was used with the “very sensitive” option, and only bacterial organisms were profiled. The abundance of antibiotic resistance genes was measured as reads per kilobase million (RPKM). Sample reads were aligned to representative sequences using bowtie2 with the sensitive-local option. The representative sequences from CARD version 2.0 (McArthur et al., 2013) were 848 ARG sequences created after clustering using cd-hit (−c 0.9 -n 8). The aligned reads remained when the aligned length was longer than 50% of the ARGs and their similarity was more than 90% matched. RPKM was considered to exist when the aligned length covered >70% of the reference length.

### Clustering, diversity, and statistical analysis

Clustering was performed according to the Dirichlet multinomial mixtures (DMM) in R package (version 4.2.0) to cluster the samples. DMM clustering was based on both genus and ARG abundance. For bacterial proportion, we included 48 genera (average proportion > 0.1% in adult or child group). The optimal number of clusters was calculated by Laplace approximation, which was selected as three and two based on the genus proportion and ARG RPKM, respectively.

Principal coordinate analysis (PCoA) was generated based on Bray–Curtis distances using the vegan package v2.5–7 in R. Alpha diversity was quantified by the Shannon index using genus proportion and ARG proportion. To measure the alpha diversity of the genus proportion, each sample was sampled as 27 M reads (4G bp size). General linear model (GLM) regression analyses were performed to validate the association between age, genus composition, and ARG composition. The PCoA and GLM regression results were compared with various labels such as age group and DMM clusters. The differential abundances of genera and ARG were determined using *t*-tests and ANOVA tests. Permutational multivariate analysis of variance (PERMANOVA) was also performed to determine the differences between each group for each genus and ARG, using the vegan package in R.

## Results

### Demographic and clinical characteristics of infants and toddlers in Korea

A total of 114 participants (53 children and 61 healthy adults) were analyzed. The children were further divided into infants (≤ 1 year, *n* = 11), toddlers (1–<4 years, *n* = 13), and school-aged children (4–13 years, *n* = 29) according to their growth stages. The age distribution of the enrolled subjects is featured in Figure 1. Clinical factors, such as delivery mode, birth weight, gestational age, and breastfeeding months for infants and toddlers, are described in Table 1. In addition, medical history, such as hospitalization, frequency of hospital visits, and use of antibiotics in the year prior to sample collection, were surveyed (data not shown). There were no statistical differences in the variables among the three groups clustered by bacterial proportion (Table 1).

**Table 1:** Groups clustered by bacterial taxonomic composition in the guts of infants, toddlers, school-aged children, and adults.

Age groups	Age definition	Number	Groups by bacterial proportion			Value of *p*
			G1	G2	G3	
Infant	≤1 year	11	0	5 (45.5)	6 (54.5)	
Toddler	1- < 4 years	13	2 (15.4)	7 (53.8)	4 (30.8)	
Child	4–13 years	29	9 (31)	11 (37.9)	9 (31)	
Adult	30–59 years	61	55 (90.2)	4 (6.6)	2 (3.3)	
Characteristics of individuals aged under <4 years						
Gender-female	*n* (%)		1 (50)	2 (16.7)	3 (30)	>0.99
BMI^a^	Median (1Q, 3Q)		15.8	17.8 (16, 18.8)	16.1 (14.2, 17.6)	0.232
Vaginal delivery	*n* (%)		2 (100)	7 (58.3)	5 (50)	0.279
Birth weight	Median (1Q, 3Q)		2.6	3.1 (1.7, 3.3)	3.1 (2.6, 3.3)	0.59
Gestational age	Median (1Q, 3Q)		37.7	37.1 (31.8, 39)	38.7 (36.1, 40.5)	0.48
Feeding month^b^						
Milk_breast	Median (1Q, 3Q)		6	3 (0, 7)	6 (3.5, 10.5)	0.27
Milk_formula	Median (1Q, 3Q)		12	11 (2, 14)	8 (5.5, 13)	0.826

a BMI, body mass index.

b Information of feeding is available in 1 in G1, 11 in G2, and 5 in G3 groups.

### Comparison of microbial composition in adults and children

**Figure 2:**
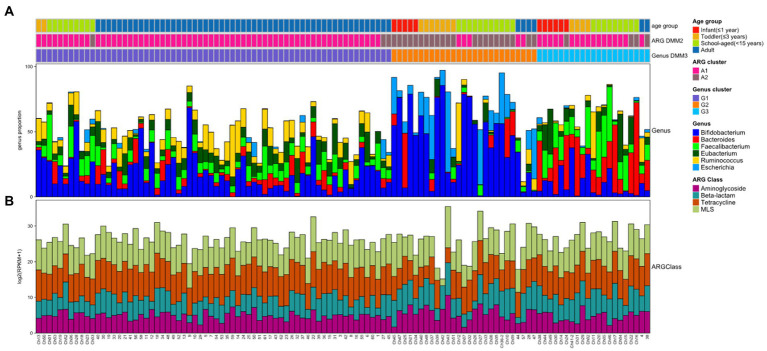
Bacterial and ARG composition in Korean adults and children. **(A)** Bacterial proportion at the genus level. The four most abundant genera are included. **(B)** Abundance of four ARG classes in each sample. The four most abundant ARG classes are included. In top bars, the top line shows the age group: infant (≤1 year) in red, toddler (≤3 years) in orange, school-aged child (≤15 years) in green, and adult in blue. The middle line shows the clusters performed by ARG abundance and DMM: A1 cluster in pink and A2 cluster in brown. The bottom line shows the clusters performed by bacterial proportion and DMM: G1 (*Ruminococcus*-*Eubacterium*-dominant group) in purple, G2 (*Bifidobacterium*-*Escherichia*-dominant group) in orange, and G3 (*Bacteroides-Faecalibacterium*-dominant group) in light blue.

The gut microbiomes of healthy adults (*n* = 61) and children (*n* = 53) were compared. Genus-level composition was quite different between adults and children (Figure 2A). The three most abundant genera in the adult group were *Bifidobacterium* (12.4%; median proportion), *Ruminococcus* (7.83%), and *Eubacterium* (7.33%), whereas the children comprised mostly of *Bifidobacterium* (24.1%), *Faecalibacterium* (4.92%), and *Bacteroides* (3.19%; Figure 2A). The samples from the child group showed two distinctive patterns: *Bifidobacterium*-dominant and *Bacteroides*-dominant. *Bifidobacterium* was the most abundant genus in one group of children and *Bacteroides* was the most abundant genus in the other group of children. To identify clusters of similar bacterial proportions, Dirichlet multinomial mixture (DMM) clustering was conducted based on the bacterial proportion. Three clusters were identified: cluster G1 was dominant in the adult samples (83.3%), and clusters G2 and G3 were dominant in child samples (85.2% and 90.5%, respectively; Figure 2A; Table 1). G1 was not found in any infants, despite their presence in toddlers and school-aged children.

**Figure 3:**
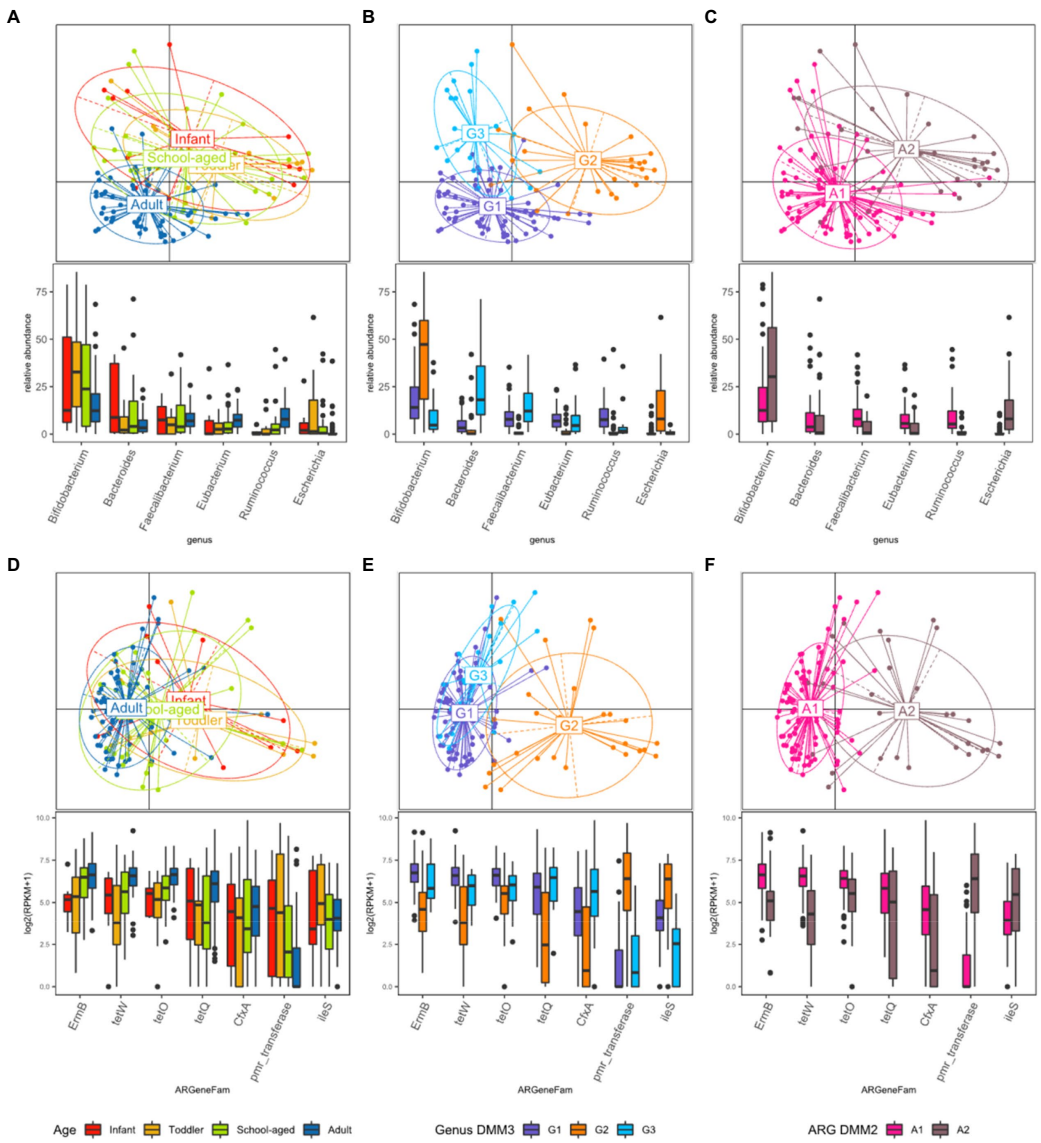
Beta diversity based on the bacterial and ARG proportion. (**A–C)** PCoA was performed with bacterial proportion at the genus level. The points are colored by **(A)** age groups, **(B)** clusters based on bacterial proportion, **(C)** clusters based on ARG families. **(D–F)** PCoA was performed with ARG families. The points are colored by **(D)** age groups, **(E)** clusters based on bacterial proportion, and **(F)** clusters based on ARG families.

PCoA was further conducted using bacterial proportion, which was labeled by the age groups and the DMM clusters of bacterial and ARG composition (Figures 3A–C). For bacterial proportion, the adult and child groups were well separated (PERMANOVA test, *p* < 0.001). In the child group, infants, toddlers, and school-aged children showed similar patterns (*p* > 0.1; Figure 3A). When the PCoA result was labeled by DMM clusters of bacterial proportion, two distinctive patterns in children were presented as G2 and G3 clusters (Figure 3B). The two most dominant genera in the G1 group were *Bifidobacterium* (12.84%, median proportion; *p* < 1.0e−8, ANOVA test) and *Ruminococcus* (7.87%; *p* < 0.005); in the G2 group were *Bifidobacterium* (47.27%; *p* < 1.0e^−8^) and *Escherichia* (7.99%; *p* < 1.0e^−9^); and in the G3 group were *Bacteroides* (18.12%; *p* < 1.0e^−9^) and *Faecalibacterium* (12.19%; *p* < 1.0e^−8^) (Figure 3B). Statistical analysis revealed significant differences in the proportions of these dominant genera among the three groups.

### Comparison of antibiotic resistance genes in adults and children

While the abundance of ARG families was measured by RPKM, the patterns of ARG proportion were compared between the age groups and clusters that were built on bacterial proportion. When DMM clustering was conducted using the ARG composition, two clusters were identified (clusters A1 and A2 in Figure 2). A1 included most adults (*n* = 56, 91.8% of adult samples; *n* = 29, 54.7% of child samples), whereas A2 included predominantly children (*n* = 24, 45.3% of child samples; *n* = 5, 8.2% of adult samples). Notably, an association was observed between the clusters of bacterial proportion (G1, G2, and G3) and ARG composition (A1 and A2). Most of the samples in G1 (95.5%) belonged to A1, and most of the samples in G2 (81.5%) belonged to A2 (Figure 2). In addition, the samples in G3, which were mostly in children, corresponded to A1. Similar clustering patterns were also observed in the PCoA (Figures 3D–F). No significant differences were observed among infants, toddlers, and school-aged children (PERMANOVA test, *p* = 0.254; Figure 3D), which was similar to the bacterial proportion (Figure 3A). However, the proportion of school-aged children (62.1%) tended to be higher in the adult-dominant A1 group than in the other child groups (45.5% and 46.2% for infants and toddlers, respectively). The dominant ARGs in A1 were *ErmB*, *tetW*, and *tetO* (*p* < 0.05, *p* < 0.001, *p* < 0.01 for A1 vs. A2, respectively), and in the A2 were *pmr transferase* and *ileS* (*p* < 0.001, *p* = 0 for A1 vs. A2, respectively; Figure 3F).

When the ARG composition was compared with the clusters based on the bacterial proportion, PCoA on ARG abundance labeled by bacterial clusters showed that the G1 and G3 groups were relatively close (Figure 3E) and mostly corresponded to A1 (Figure 3E,F). Among the children, the samples included in G2 showed a higher abundance of *Bifidobacterium* intrinsic *ileS* and *pmr transferase* compared to the samples in G3 (Figure 3E). These two genes, *ileS* and *pmr transferase*, are frequently found in *Bifidobacterium* and *Escherichia*, respectively (Lee et al., 2004; Serafini et al., 2011), which were the most abundant genera in G2. In contrast, the samples in G3 showed a higher abundance of *ErmB*, tetW, *tetO*, *tetQ*, and *CfxA*, which are commonly identified in anaerobic gut flora (Figure 3E)^1^ (Parker and Smith, 1993; Scott et al., 2000; Melville et al., 2001).

### Compositional changes in microbes and antibiotic resistance genes with aging

**Figure 4:**
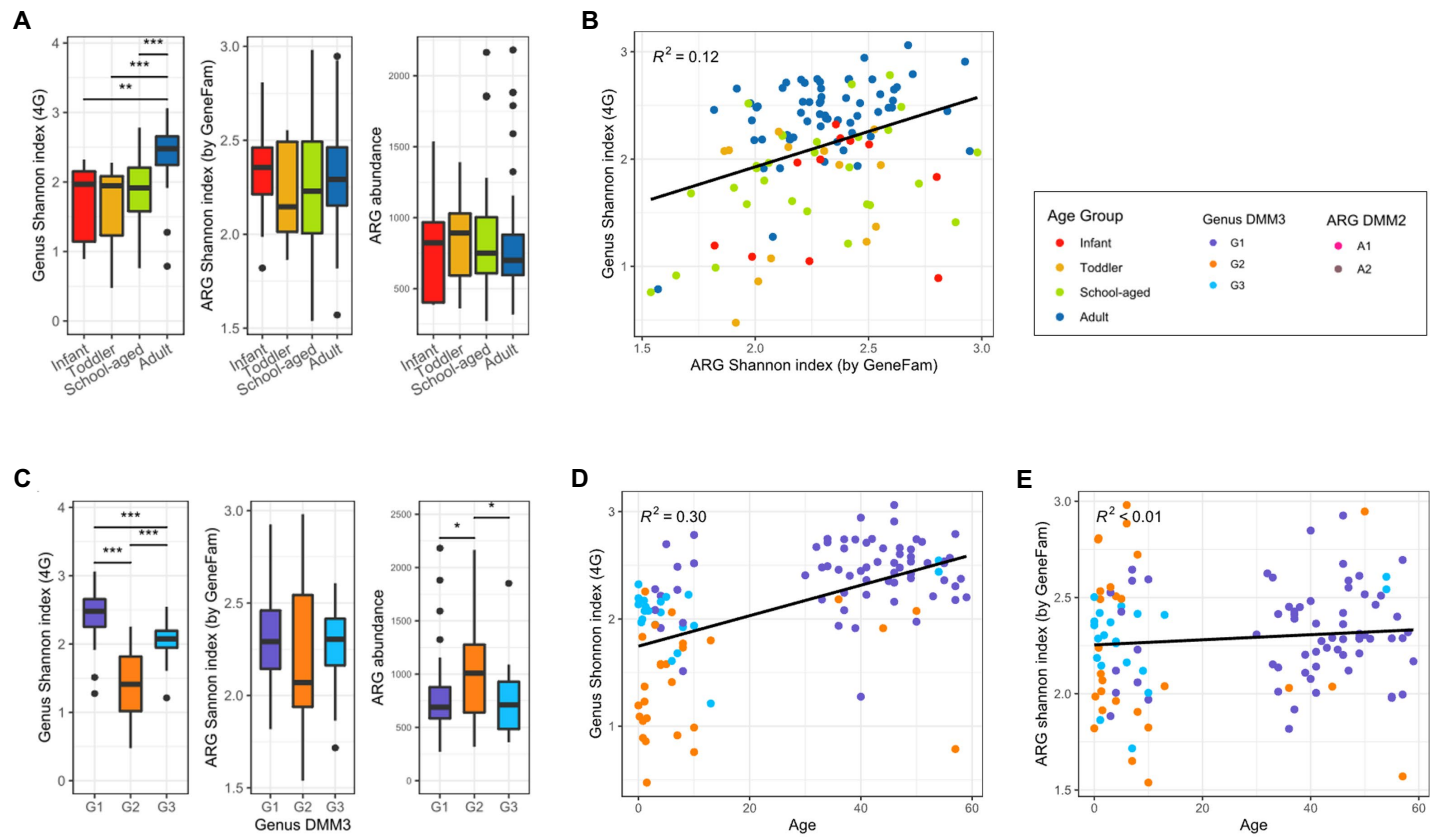
Bacterial and ARG diversity in adults and children. **(A)** Alpha diversity of bacterial and ARG composition, and ARG abundance in four age groups. **(B)** Correlation between bacterial and ARG diversity. **(C)** Alpha diversity of bacterial and ARG composition, and ARG abundance in three clusters based on bacterial proportion. **(D)** Bacterial diversity over age. **(E)** ARG diversity over age. Significant differences of all boxplots were denoted as follows: *p < 0.05, ** *p* < 0.01, ***p < 0.001; *t*-test).

Bacterial and ARG compositions were compared to determine their association and to examine how microbial and ARG compositions changed with age. Compared to the child samples, bacterial diversity was higher in the adult samples (Figure 4A). However, there was almost no difference among infants, toddlers, and school-aged children (median Shannon index: 1.97, 1.95, and 1.92, respectively; Figure 4A). Accordingly, the adult-dominant cluster G1 had the highest genus diversity, followed by G3 and G2 (2.48, 2. 08, and 1.41 for G1, G3, and G2, respectively; median Shannon index). In addition, alpha diversity was positively correlated with age (*R*^2^ = 0.3; Figures 4C,D). However, as age increased, the ARG diversity did not change significantly (*R*^2^ < 0.01; Figure 4E). ARG diversity in infants and adults was slightly higher than that of toddlers and school-aged children without statistical significance (2.36, 2.29, 2.15, and 2.23 in infants, adults, toddlers, and school-aged children; median Shannon index) (Figure 4A). Among the DMM clusters of bacterial proportion, the G2 group showed both the least diversity of ARGs and the most abundant ARGs (Figure 4C). Notably, as ARG diversity increased, bacterial diversity increased (*R*^2^ = 0.12) (Figure 4B). ARG diversity was not correlated with ARG abundance (*R*^2^ < 0.01).

**Figure 5:**
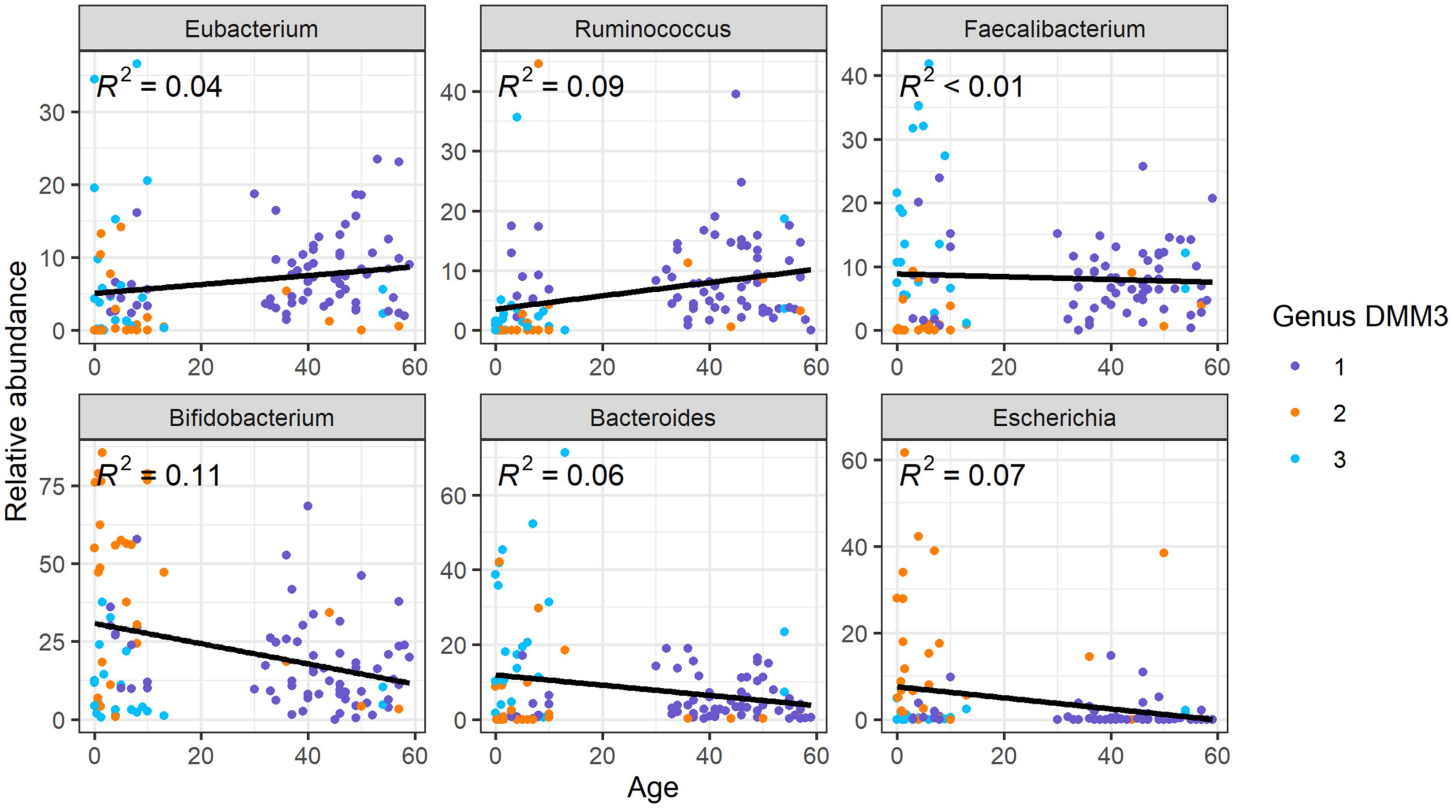
Correlation between relative abundance of the gut microbiota at the genus level and age.

Upon comparison of the proportions of specific genera and ARGs to age, we found that the *Eubacterium* and *Ruminococcus*, which were the dominant genera in G1 (adult-dominant cluster), showed positive correlations (*R*^2^ = 0.04 and 0.09, respectively) with age (Figure 5). However, *Bifidobacterium*, *Bacteroides*, and *Escherichia*, which were the major genera in G2 or G3 (child-dominant clusters) (*p* < 0.05 between adults and children), showed a negative correlation (*R*^2^ = 0.11, 0.06, and 0.07, respectively) with age (Figure 5). Notably, *Bifidobacterium* showed the strongest correlation (*R*^2^ = 0.11) with age.

**Figure 6:**
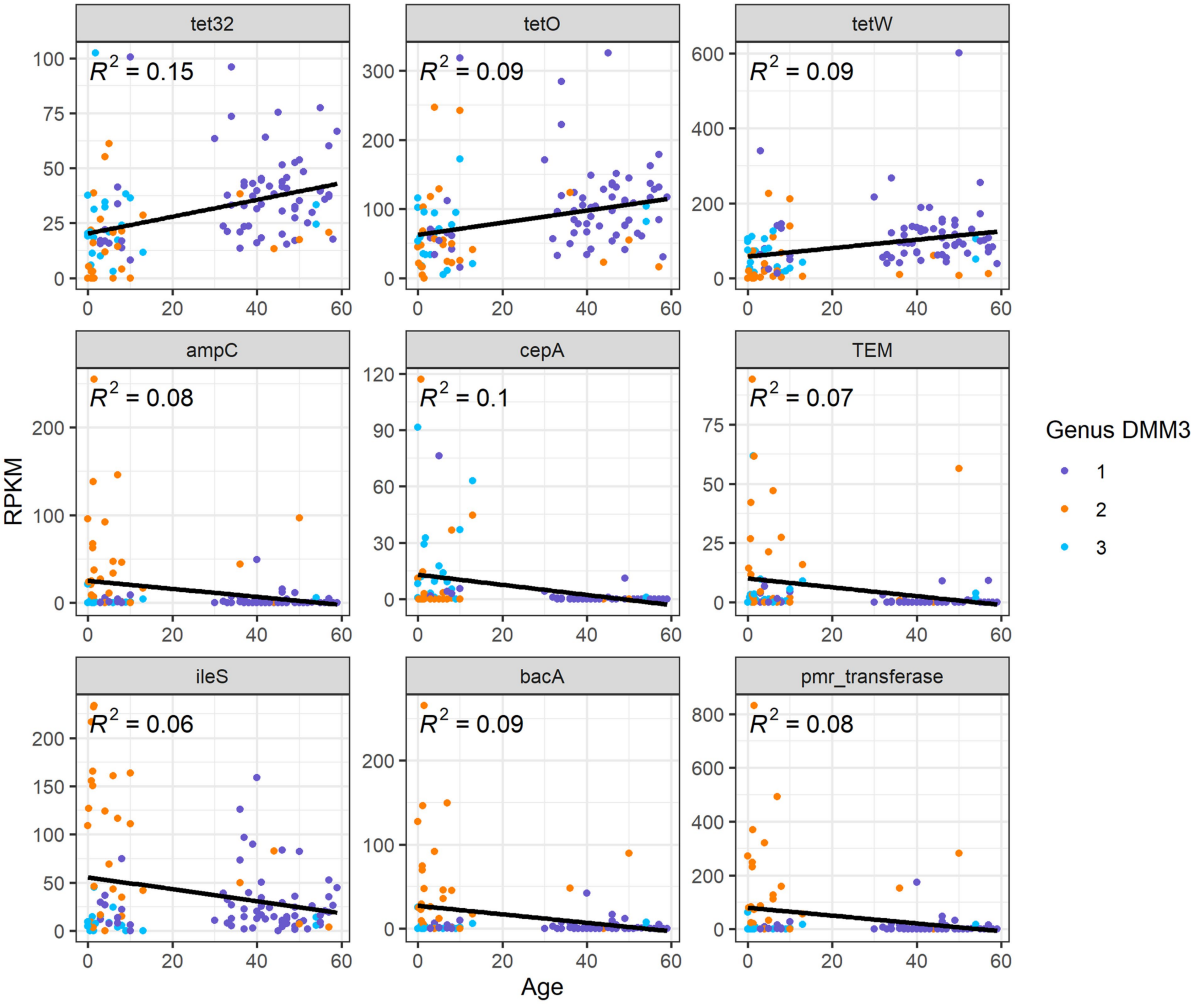
Correlation between ARG abundance and age.

Overall, nine ARG families showed significant differences among age groups or clusters based on bacterial proportion (*p* < 0.001 in any pair of groups; Figure 6). Furthermore, three tetracycline resistance genes, tet32, tetO, and tetW, were positively correlated (*R*^2^ = 0.15, 0,09, and 0.09, respectively) with age (Figure 6). However, six other genes, *ampC*, *TEM*, *ileS*, *bacA*, *pmr transferase*, and *cepA*, showed a negative correlation (*R*^2^ = 0.08, 0.07, 0.06, 0.09, 0.08, and 0.1, respectively) with age (Figure 6).

## Discussion

Many studies have monitored the dynamics of the gut microbiota during the first 3 years of life in humans and have revealed common patterns of development across countries. Bacterial alpha diversity increases with age, whereas interindividual variations decrease (Derrien et al., 2019). *Bifidobacterium* enterotypes in infancy mature into stable *Bacteroides* and *Prevotella* enterotypes with aging (Bäckhed et al., 2015; Gibson et al., 2016; Stewart et al., 2018; Derrien et al., 2019; Xiao et al., 2021). *Bifidobacterium* and *Bacteroides* enterotypes were observed consistently in the first 3 years of life, but the *Prevotella*-dominant enterotype did not appear until the second year of life (Xiao et al., 2021).

In this population, *Bifidobacterium* enterotype persisted until late childhood. This delayed persistence of *Bifidobacterium*-dominant enterotype was similarly reported in Asian, Dutch, and South American countries (Nakayama et al., 2015; Zhong et al., 2019; Xiao et al., 2021). The proportion of *Bifidobacterium* was relatively high in G1, which was found in most adult populations in this study. The high abundance of *Bifidobacterium* in the adult population may be associated with the delayed persistence of G2 in children. A study based on a Japanese population revealed a similar finding; the gut of healthy Japanese adults showed a higher abundance of the phylum *Actinobacteria*, particularly the genus *Bifidobacterium* (Nishijima et al., 2016), while school-aged Japanese children had a higher relative abundance of *Bifidobacterium* with a lower alpha diversity in their gut than those children from other countries, such as China, Taiwan, Thailand, and Indonesia (Nakayama et al., 2015). In the rural Tyva region of Siberia in Russia, butyrate-producing Firmicutes enterotype with a high proportion of *Bifidobacterium* was identified, which differed from the microbiota of people from urban area in Russia (Koecher et al., 2015). Tyva are Turkic-speaking ethnic group mainly residing in the remote republic on the Russian-Mongolian border (Health Care Utilisation, 2016).

Diet and lifestyle were considered to impact shaping gut microbiota (De Filippo et al., 2010; Gorvitovskaia et al., 2016; Zhong et al., 2019). The gut microbiome of people from Western countries having a Western diet tends to be dominated by *Bacteroides* and Clostridiales, while rural populations with a high-fiber, low-protein diet tend to be dominated by *Prevotella* (29). Diet in Korea contains much sugar, animal fat, and calorie-rich food but little plant fiber, therefore *Prevotella* enterotype was rarely present in children and adults in Korea. Several studies have suggested an association between the *Bifidobacterium*-enriched enterotype and diet. In Dutch preschool children, less than average dietary fiber intake was associated with the *Bifidobacterium*-rich enterotype (Zhong et al., 2019). A previous study reported a significantly lower abundance of *Bifidobacterium* in healthy subjects on a fiber-blend formula diet than in subjects on a fiber-free formula diet (Koecher et al., 2015). Functional analyses demonstrated that the gut of children enriched with *Bifidobacterium* strains favored the utilization of simple sugars but lacked the potential for complex carbohydrate utilization (Zhong et al., 2019). Antibiotic usage in infants and toddlers might affect the delayed persistence of G2. Korea has the highest healthcare utilization *per capita* among the OECD countries (Health Care Utilisation, 2016), and frequent visits to outpatient clinics might lead to increased use of antibiotics in infants and toddlers, and the early-life exposure of antibiotics has been previously shown to affect the composition of gut microbiota and ARGs (Li et al., 2021; Lebeaux et al., 2022). Consequently, the delayed persistence of the *Bifidobacterium*-dominant enterotype makes it subject to further research to understand its persistence.

The gut resistome is known to be associated with several factors: age of the infant, type of delivery, breast versus formula feeding, gestational age, and intrapartum antibiotic usage (Bäckhed et al., 2015; Gibson et al., 2016; Lebeaux et al., 2021; Li et al., 2021; Lebeaux et al., 2022). Generally, the abundance of ARGs in the infant gut has been shown to decrease with age (Bäckhed et al., 2015; Gibson et al., 2016). The taxonomic signature in gut microbiota is associated with the resistome (Gibson et al., 2016; Lebeaux et al., 2021; Li et al., 2021; Lebeaux et al., 2022), and the overall relative abundance of the resistome was strongly correlated with *Proteobacteria*, specifically *Escherichia coli (*Lebeaux et al., 2021*)*. In the present study, the G2 group with lower bacterial diversity showed a lower ARG diversity, but a higher ARG abundance because of probably the high relative abundance of *Escherichia* in the group.

This study highlights its focus on the gut microbiome of infants and children in the Korean population, providing novel insights into their bacterial proportion which is unlike the gut composition of those described in studies based on European populations. Furthermore, the results of this study revealed a delayed persistence of the *Bifidobacterium* enterotype, suggesting a maturation process with different bacterial proportion and delayed maturation times. However, we did not follow up each individual infant longitudinally; thus, the development process of the gut microbiota could not be observed directly. Moreover, the number of subjects in each age group was small for generalization.

In summary, the bacterial proportion of the gut in the population of Koreans ranging from infants to adults formed three clusters: the *Ruminococcus*-*Eubacterium* group (G1), *Bifidobacterium*-*Escherichia* group (G2), and *Bacteroides*-*Faecalibacterium* group (G3). Infants and children under 3 years of age showed mostly G2 and G3, but 90% of adult showed G1 type gut microbiota, whereas the children in the age group between 4 and 13 years showed transitional features. The diversity of genera and ARGs was the lowest in G2, but interindividual diversity of genera and relative abundance of ARGs were the highest in G2.

## Data availability statement

The datasets presented in this study can be found in online repositories. The European Nucleotide Archive (ENA) and PRJEB33013 and PRJEB55713 can be found at: https://www.ebi.ac.uk/ena/browser/view/PRJEB33013 and https://www.ebi.ac.uk/ena/browser/view/PRJEB55713.

## Ethics statement

The studies involving human participants were reviewed and approved by the study protocol was approved by the Institutional Review Board (HYUH IRB 2019–09-030) and written informed consent was obtained from all the participants. Written informed consent to participate in this study was provided by the participants’ legal guardian/next of kin.

## Author contributions

MR, SH, and HP: conceptualization and funding acquisition. JieK, BK, JinK, and HL: data curation. JieK, EK, BK, JinK, J-SP, and HL: investigation. EK and MR: methodology. JieK, EK, MR, and HP: writing: original draft. JinK, MR, and HP: writing: review and editing. All authors contributed to the article and approved the submitted version.

## Funding

This study was supported by a grant from the National Medical Center, Republic of Korea (grant number: NMC2019-MS-05).

## Conflict of interest

Author JinK is employed by Novel Pharma Inc.

The remaining authors declare that the research was conducted in the absence of any commercial or financial relationships that could be construed as a potential conflict of interest.

## Publisher’s note

All claims expressed in this article are solely those of the authors and do not necessarily represent those of their affiliated organizations, or those of the publisher, the editors and the reviewers. Any product that may be evaluated in this article, or claim that may be made by its manufacturer, is not guaranteed or endorsed by the publisher.

## Footnotes

^1^ https://card.mcmaster.ca/ontology/39435/
